# Personalized Antithrombotic Strategies in Patients with Atrial Fibrillation Following Transcatheter Aortic Valve Replacement

**DOI:** 10.3390/jpm15040149

**Published:** 2025-04-09

**Authors:** Razan Awan, Monirah A. Albabtain, Aisha AlRasheedi, Maha AlHarthi, Zaid Alanazi, Amr A. Arafat

**Affiliations:** 1 Pharmacy Department, Prince Sultan Cardiac Center, Riyadh 12231, Saudi Arabia; razan.awan@gmail.com (R.A.); aishamutie@gmail.com (A.A.); mmharthi.9@gmail.com (M.A.); zalanazi@pscc.med.sa (Z.A.); 2 King Fahad Armed Forces Hospital, Jeddah 23311, Saudi Arabia; 3 Research Department, Prince Sultan Cardiac Center, Riyadh 12231, Saudi Arabia; 4 Pharmacy Department, King Fahad Specialist Hospital, Buraydah 52366, Saudi Arabia; 5 Health Research Center, Ministry of Defense Healthcare Services, Riyadh 12485, Saudi Arabia; amr.arafat@med.tanta.edu.eg; 6 Adult Cardiac Surgery Department, Prince Sultan Cardiac Center, Riyadh 12231, Saudi Arabia; 7 Cardiothoracic Surgery Department, Tanta University, Tanta 31111, Egypt

**Keywords:** transcatheter aortic valve replacement, antiplatelet, anticoagulants, direct oral anticoagulants, stroke, bleeding

## Abstract

**Background:** Atrial fibrillation (AF) is prevalent in patients undergoing transcatheter aortic valve replacement (TAVR). However, the optimal antithrombotic strategy tailored to individual patient profiles remains unclear. This study aims to evaluate the outcomes of personalized antithrombotic regimens in patients with AF after TAVR. **Methods:** We enrolled 121 AF patients who underwent TAVR from 2009 to 2023. Patients were grouped into seven groups based on individualized post-procedural antithrombotic regimens. The regimens included the following: single antiplatelet therapy (SAPT) + direct oral anticoagulant (DOAC) (n = 44, 36.3%); DOACs only (n = 25, 20.6%), SAPT + warfarin (n = 17, 14%); dual antiplatelet therapy (DAPT) (n = 13, 10.7%); warfarin only (n = 8, 6.6%); DAPT + warfarin (n = 7, 5.8%); and DAPT + DOACs (n = 7, 5.8%). The study outcomes included incidences of strokes or transient ischemic attacks (TIAs), major bleeding, and survival. **Results:** The median follow-up was 27 months. The incidence of stroke, TIA, or major bleeding was similar among the seven treatment groups. However, a trend toward a higher rate of stroke was observed in the triple regimen containing warfarin (28.6%); also, the highest rate of major bleeding was observed in the warfarin-only group (25%). Survival for patients discharged and placed under various antithrombotic regimens did not differ significantly despite some numerical variations being present across the groups, with the lowest mortality reported with SAPT + warfarin (7%) and the highest with DAPT + warfarin (57%). **Conclusions:** This study highlights the outcomes related to stroke, major bleeding, and mortality across personalized antithrombotic regimens in patients with AF after TAVR. While no statistically significant differences were observed, findings emphasize the need for further large-scale studies to define optimal personalized antithrombotic strategies based on individual patient characteristics.

## 1. Introduction

Transcatheter aortic valve replacement (TAVR) has become the cornerstone therapy for symptomatic severe aortic stenosis patients. With advancements in device technology and operator experience, TAVR is now recommended for older patients with aortic stenosis suitable for transfemoral approaches [1,2,3,4]. Atrial fibrillation (AF) is prevalent in more than one-third of TAVR patients, with new-onset AF occurring in up to 36% of post-procedures [5]. The presence of AF—whether preexisting or newly developed—significantly raises the risk of mortality within one year and increases the likelihood of stroke and bleeding, particularly in TAVR patients [6,7,8].

In the context of personalized medicine, the CHA2DS2-VASc score serves as a critical tool for estimating individual risks of stroke, transient ischemic attack, and systemic embolism in patients with AF undergoing TAVR. This score guides the recommendations for oral anticoagulant (OAC) therapies, particularly for patients with a score of one or more in men or two or more in women [1]. The ESC/EACTS guidelines advocate for lifelong OAC therapy post-TAVR, unless the patient has recently undergone a percutaneous coronary intervention (PCI), in which case, dual therapy with OAC and aspirin or clopidogrel is suggested for 1–6 months, followed by lifelong anticoagulation therapy [1]. Conversely, the ACC/AHA guidelines lack specific recommendations for this patient population [2].

Research by Kosmidou and colleagues assessed the efficacy of OAC alone for stroke prevention after TAVR in the PARTNER 2 cohort study, revealing that OAC alone did not significantly lower stroke incidence over two years. However, antiplatelet therapy—whether combined with or without anticoagulant therapy—did show a reduction in stroke risk at two years. OAC alone was associated with a decreased risk of combined death and stroke [9]. Notably, combining OAC therapy with antiplatelet therapy resulted in increased bleeding complications without a corresponding benefit in long-term thromboembolic risk reduction [10].

Current clinical practice for antithrombotic therapy in patients with atrial fibrillation (AF) undergoing TAVR often does not reflect the principles of personalized medicine. Given the variability in bleeding risks, stroke rates, and mortality among these patients, this study aims to evaluate the effectiveness of various antithrombotic regimens tailored to individual patient profiles, including genetic predispositions and comorbid conditions, in those undergoing TAVR with preexisting or new-onset AF.

## 2. Methods

### 2.1. Design and Patients

**Figure 1:**
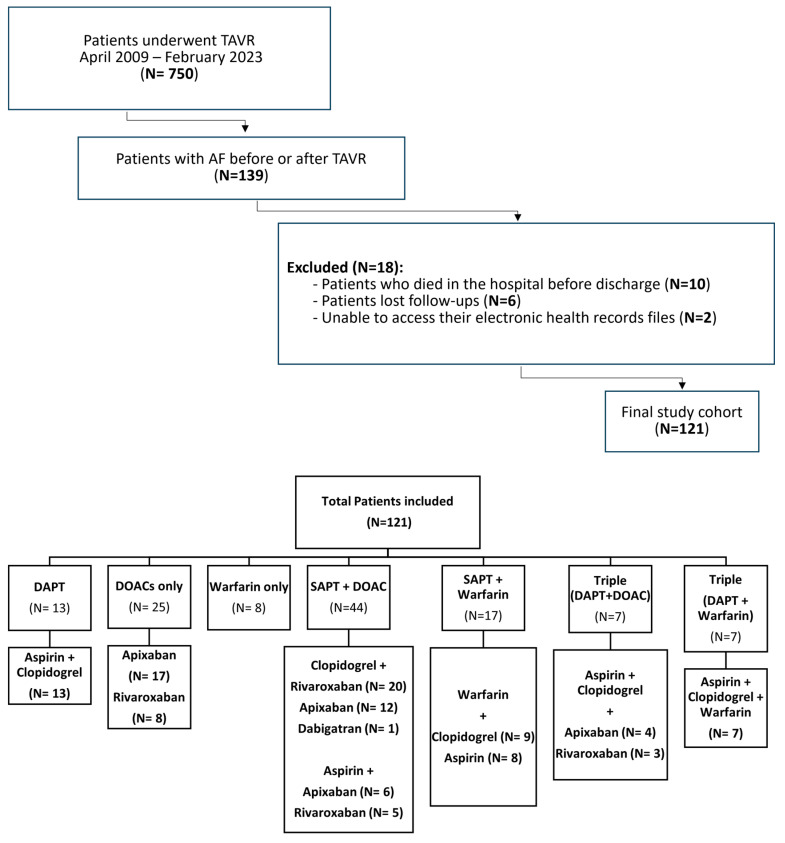
Study flowchart of patients with atrial fibrillation and transcatheter aortic valve replacement who were discharged on antithrombotic. AF: atrial fibrillation; TAVR: transcatheter aortic valve replacement; DAPT: dual antiplatelet; SAPT: single antiplatelet; DOACs: direct oral anticoagulants.

This retrospective observational cohort study was conducted at a tertiary cardiac center. We included all consecutive patients with atrial fibrillation (AF) or those who developed new-onset AF after transcatheter aortic valve replacement (TAVR) (n = 121) and were discharged on antithrombotic therapy from April 2009 to February 2023. All patients who underwent TAVR during the study period were assessed for individual risk factors and clinical characteristics to inform personalized treatment strategies. Based on these assessments, patients were categorized into the following seven groups according to their tailored antithrombotic regimens: single antiplatelet therapy (SAPT) + direct oral anticoagulant (DOAC) (n = 44, 36.3%); DOACs only (n = 25, 20.6%); SAPT + warfarin (n = 17, 14%); dual antiplatelet therapy (DAPT) (n = 13, 10.7%); warfarin only (n = 8, 6.6%); DAPT + warfarin (n = 7, 5.8%); and DAPT + DOACs (n = 7, 5.8%). The study flowchart, grouping, and medications are presented in Figure 1.

This study was approved by the Prince Sultan Military Medical City Institutional Review Board (Approval number: 1681, date 2 October 2023). The need for patient consent was waived due to the retrospective design of this study.

### 2.2. Data and Endpoints

Data were collected from the hospital’s electronic records and used to populate a REDCap project designed for this research. The study outcomes included stroke, TIAs, major bleeding events, and survival during follow-up, with an emphasis on how personalized treatment plans impact these outcomes. The Valve Academic Research Consortium-2 (VARC-2) definitions were used for bleeding complications, defining major bleeding as a decrease in hemoglobin of 3 g/dL or requiring a transfusion of 2–3 units [11]. Patients who were lost to follow-up or who died in the hospital were excluded. All patients were followed in the post-TAVR clinic every 6–12 months, with personalized follow-up plans based on their specific antithrombotic regimen. Patients were instructed to report any bleeding or neurological symptoms to the clinic or the emergency department.

### 2.3. Statistical Analysis

Data are presented as medians, interquartile ranges, and percentages for patients’ baseline characteristics. Categorical variables were compared via the chi-square test or Fisher’s exact test, whereas quantitative variables were compared via the Kruskal-Wallis test. The time-to-event variable distribution was plotted via Kaplan-Meier curves. Time-to-event outcomes were compared via the log-rank test. Multivariable Cox regression was performed for stroke and survival, adjusting for baseline coronary artery disease and peripheral arterial disease. A trend test was performed using ranks for the response scores in the test due to Cuzick. All analyses were performed via Stata 16 (Stata Corp., College Station, TX, USA), and a *p*-value of less than 0.05 was considered statistically significant.

## 3. Results

### 3.1. Baseline Data

This study analyzed various personalized antithrombotic regimens and their outcomes among different patient groups. Most baseline characteristics were not significantly different among these groups. However, the prevalence of atrial fibrillation (AF), coronary artery disease (CAD), and prior percutaneous coronary intervention (PCI) were significantly higher in the DAPT + warfarin group (*p* = 0.035, *p* < 0.0001, *p* = 0.018, respectively). Conversely, peripheral arterial disease (PAD) was significantly more common in the SAPT + warfarin group (47%) (*p* = 0.001). There were trivial differences in the CHA2DS2-VASc or HAS-BLED scores among the groups, indicating uniformity in assessing stroke and bleeding risks. There was a trend toward using NOAC in recent years (*p* = 0.047) (Table 1).

### 3.2. Procedure Data

Patients requiring concomitant PCI were more frequently assigned a triple therapy (DAPT + DOAC), reflecting a tailored approach to antithrombotic management based on individual procedural needs. There were no differences in other procedural details among these groups (Table 2).

### 3.3. Hospital Outcomes

Hospital outcomes were compared among patients with different personalized antithrombotic regimens, showing similar incidence in early myocardial infarction, permanent pacemaker insertion, stroke, or bleeding events (Table 3).

### 3.4. Follow-Up Outcomes

The median follow-up was 27 months (25th–75th percentiles: 11–55). Regarding primary outcomes, the incidence of stroke was highest in the triple regimen containing warfarin (28.6%); however, there was similarity in stroke incidence among the seven groups. Major bleeding rates were also comparable among the groups, with the highest rate being observed in the warfarin-only group (25%). Mortality during follow-up occurred in 24 patients, and it was greater in the triple therapy (DAPT + warfarin) group (57.14%) (Table 4).

**Figure 2:**
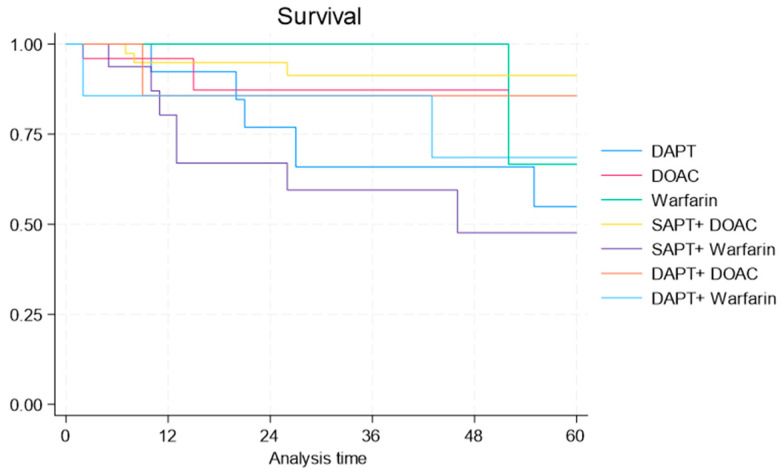
Kaplan-Meier curves for survival in patients with atrial fibrillation after transcatheter aortic valve replacement receiving different antithrombotic regimens. DAPT= dual antiplatelet; SAPT= single antiplatelet; DOACs = direct oral anticoagulants.

Survival analysis indicated that the triple therapy groups, namely DAPT + DOACs and DAPT + warfarin, experienced the greatest decline in survival over 5 years, with the DAPT + warfarin group exhibiting the poorest overall survival. In unadjusted analyses, there were unremarkable differences in survival rates for patients discharged on various personalized antithrombotic regimens (Figure 2).

Multivariable Cox regression for stroke adjusting for PAD and CAD revealed a negligible effect for the treatment group (HR: 0.84, 95% CI: 0.62–1.13). Similarly, the groups did not affect survival (HR: 1.11, 95% CI: 0.87–1.42).

## 4. Discussion

Finding the optimal balance between preventing ischemic events and minimizing bleeding risk with post-TAVR antithrombotic therapy remains a significant clinical challenge that requires further investigation, particularly in the context of personalized medicine. Personalized medicine in this context involves tailoring antithrombotic regimens to the unique clinical profiles of individual patients, taking into account factors such as age, comorbidities, and specific risk factors for both thromboembolic and bleeding complications. By leveraging advancements in patient stratification, clinicians can better assess the risks and benefits of various antithrombotic strategies. This individualized approach aims to optimize therapeutic outcomes while minimizing adverse effects, thereby enhancing patient safety and overall care quality. As our understanding of the interactions between patient characteristics and medication responses evolves, the implementation of personalized medicine could significantly improve the management of AF patients undergoing TAVR, ensuring that each patient receives the most appropriate and effective antithrombotic therapy. Several clinical trials have evaluated the addition of antiplatelet therapy to anticoagulation treatment in patients undergoing TAVR who have a concurrent indication for anticoagulation. Findings from these clinical trials suggest that patients with preexisting indications for anticoagulation should ideally maintain anticoagulation therapy alone, as the addition of antiplatelet therapy is linked to an increased risk of bleeding complications without significant benefits in reducing ischemic events [9,12].

This real-world study provides insights into the variety of personalized antithrombotic regimens prescribed for patients with AF undergoing TAVR in our institution, revealing the use of seven distinct regimens that include both anticoagulants and antiplatelet agents. These findings highlight the clinical challenge of balancing bleeding and clotting risks in a patient population that represents approximately 18% of total TAVR cases. The significant variability in discharge antithrombotic regimens indicates a lack of consensus on the optimal treatment approach. Notably, 10.7% of patients were discharged on dual antiplatelet therapy without oral anticoagulants, underscoring the need for personalized strategies tailored to individual patient profiles. When comparing our findings with those of Sherwood et al., we observed differences in discharge medication regimens, with a smaller fraction of patients (6.7%) receiving oral anticoagulants alone, whereas a much higher percentage in their study (51.2%) received oral anticoagulants alongside antiplatelets [13]. Moreover, the study by Altisent and collaborators included mostly patients (84%) on oral anticoagulants and antiplatelets [12]. These varying patterns in practice may stem from localized guidelines and clinical practices. The best antithrombotic strategies for patients at increased risk of both thromboembolic and bleeding complications remain unclear [1,2].

Expert consensus guidelines differ, with American recommendations suggesting the use of warfarin, direct thrombin inhibitors, or factor Xa inhibitors in conjunction with low-dose aspirin [14], while European guidelines advocate for warfarin with aspirin or thienopyridine [15]. Canadian guidelines favor the use of DOACs whenever possible, reflecting the inconsistency in recommendations that may confuse practitioners due to insufficient research supporting management decisions [16]. Currently, optimal regimens are being investigated in several randomized controlled trials. A crucial revelation of underusing oral anticoagulants in high-risk patients, such as those who undergo TAVR with AF, has important implications [13].

Among our patient population, the median CHA2DS2-VASc score was 4, with 71% scoring ≥4, demonstrating a pressing need for oral anticoagulants, which aligns with Sherwood et al.’s findings, in which 75% of patients had a CHADS2-VASC score of ≥4 [13]. Decisions can be complex, particularly for patients with concurrent indications for dual antiplatelet therapy, such as CAD and PCI; however, this represented only a minority of our study population. Concerns about bleeding risks or underestimating stroke risks may lead to underuse of oral anticoagulants in some AF patients [13,17]. Studies indicate that physicians often do not adequately assess bleeding risk, resulting in unnecessary caution [18]. Several organizations have developed strategies to enhance adherence to guideline recommendations for patients with atrial fibrillation (AF) who are at moderate to high risk of stroke [19,20]. As a result of TAVR, stroke remains a very common, devastating complication; moreover, patients with AF who undergo TAVR are considered at increased risk of stroke [13].

Recent studies have investigated the efficacy and safety of DOAC or warfarin in patients with AF undergoing TAVR. In the ENVISAGE-TAVI AF trial [21], edoxaban was noninferior to warfarin in patients with preexisting AF who underwent TAVR, as measured by a hazard ratio margin of 38% for the primary composite outcomes of adverse events. However, major bleeding was greater with edoxaban than with warfarin. Furthermore, apixaban was not superior to the standard of care in the ATLANTIS trial [22], regardless of anticoagulation indications. The AVATAR trial is another ongoing trial that investigates the efficacy and safety of anticoagulation alone versus anticoagulation and aspirin following TAVR (NCT02735902). Having access to these data may assist clinicians in understanding the use of anticoagulants following TAVR and illustrating the importance of oral anticoagulants in these high-risk patients.

We reported no differences in outcomes across the groups concerning stroke rates, bleeding events, or mortality. However, stroke and mortality rates were numerically greater in the DAPT + warfarin group, and major bleeding events were highest in the warfarin-only group. Our findings partly align with those of Sherwood et al.’s study [13], which included 11,382 patients. Their study also revealed that the use of oral anticoagulants + antiplatelets did not reduce the risk of stroke or all-cause mortality compared with the use of oral anticoagulants alone or antiplatelets alone; however, there are some conflicts between our results and their findings. Specifically, Sherwood and associates [13] reported an increased bleeding risk with combination therapy compared with antiplatelet therapy alone, whereas we reported that the bleeding risk was similar between combination therapy and oral anticoagulant therapy alone. Additionally, Altisent and collaborators [12] conducted a small multicenter study of 621 patients and demonstrated no superiority of combining oral anticoagulants and antiplatelet therapy; however, the risk of bleeding increased.

In contrast, in the randomized POPULAR-TAVI trial [23], 326 patients with AF who underwent TAVR were randomized to oral anticoagulant alone versus oral anticoagulant + clopidogrel. There was a significant reduction in major bleeding with oral anticoagulants alone (21.7% vs. 34.6%; risk ratio, 0.63; 95% CI, 0.43 to 0.90; *p* = 0.01), whereas there was no significant difference in the rates of death from cardiovascular causes and ischemic events compared with oral anticoagulants + clopidogrel.

In our unadjusted analyses and those adjusted for coronary artery disease and peripheral arterial disease, we observed no differences in 5-year survival rates among patients discharged on various antithrombotic regimens. This result was consistent with the findings of the PARTNER II trial and associated registries, which revealed that patients who received oral anticoagulants, antiplatelets, or a combination of both did not differ in the combined endpoint of death or stroke after two years [9].

Antithrombotic regimens for patients with AF undergoing TAVR have not been compared in prospective studies. In addition, current guidelines rely on empirical evidence. Considering the available evidence, prospective randomized studies comparing different strategies involving anticoagulants alone and in combination with antiplatelets are imperative.

### Strengths and Limitations

Our study offers valuable insights into the long-term cardiovascular effects of different antithrombotic regimens from a clinically relevant perspective. However, the observational study design has several limitations. On the positive side, this design enabled this study to reflect real-world practice. Unfortunately, the small sample size in each group limits the ability to extensively extrapolate our results and limits their generalizability. This study covers data from 2009 to 2023, which means that it takes into account the evolution of antithrombotic treatments and the growing adoption of DOACs. Furthermore, this study presents a single-center experience, and generalizing the results to other institutions could not be possible. Notably, our study uniquely focused on patients with preexisting AF and those who developed new-onset AF within 30 days of undergoing TAVR. Another limitation is the heterogeneity of the patient population, which may have influenced the observed outcomes.

## 5. Conclusions

Although this study found no significant differences in stroke, major bleeding, or mortality among the various antithrombotic regimens in patients with AF who underwent TAVR, the differing rates of primary outcomes across these regimens underscore the importance of personalized medicine. Clinicians should carefully consider the choice of antithrombotic therapy based on individual patient profiles, including factors such as comorbidities, genetic predispositions, and risk factors for both thromboembolic and bleeding complications. To optimize patient outcomes, further studies are needed to define the most effective and safe antithrombotic strategies tailored specifically for AF patients post-TAVR, ultimately enhancing the quality of care in this high-risk population.

## Figures and Tables

**Table 1:** Baseline and clinical characteristics of patients with atrial fibrillation and transcatheter aortic valve replacement treated with different antithrombotic regimens.

	DAPT N = 13	DOAC Only N = 25	Warfarin Only N = 8	SAPT + DOAC N = 44	SAPT + Warfarin N = 17	Triple (DAPT + DOAC) N = 7	Triple (DAPT + Warfarin) N = 7
Age (years)	81 (72–87)	76 (70–81)	75 (68–78.5)	75 (69–81)	72 (68–79)	72 (68–89)	74 (68–80)
Male	9 (69.23%)	11 (44%)	4 (50%)	22 (50%)	6 (35.29%)	5 (71.43%)	4 (57.14%)
Body mass index (kg/m^2^)	30.82 (26.6–33.7)	30.08 (27.5–35.1)	28.55 (27.5–29.5)	30.565 (26.1–36.5)	32.88 (28.4–35.6)	28.05 (25–33.6)	26.54 (22.5–34.6)
Hypertension	10 (76.92%)	22 (88%)	6 (75%)	35 (79.55%)	14 (82.35%)	7 (100%)	5 (71.43%)
Diabetes	9 (69.23%)	18 (72%)	5 (62.50%)	26 (59.09%)	11 (64.71%)	5 (71.43%)	4 (57.14%)
Dyslipidemia	6 (46.15%)	16 (64%)	4 (50%)	18 (40.91%)	5 (29.41%)	4 (57.14%)	2 (28.57%)
Hypothyroidism	1 (7.69%)	9 (36%)	1 (12.50%)	5 (11.36%)	1 (5.88%)	0	1 (14.29%)
Anemia	4 (30.77%)	4 (16%)	2 (25%)	14 (31.82%)	4 (23.53%)	1 (14.29%)	1 (14.29%)
Dialysis	1 (7.69%)	0	0	1 (2.27%)	1 (5.88%)	0	0
Chronic Liver disease	1 (7.69%)	1 (4%)	1 (12.50%)	0	1 (5.88%)	0	0
Chronic lung disease	3 (23.08%)	6 (24%)	2 (25%)	8 (18.18%)	5 (29.41%)	1 (14.29%)	3 (42.86%)
Cancer within 5 years	1 (7.69%)	1 (4%)	0	4 (9.09%)	1 (5.88%)	0	0
Cerebrovascular disease	2 (15.38%)	0	1 (12.50%)	6 (13.64%)	3 (17.65%)	0	1 (14.29%)
Coronary artery diseases	9 (69.23%)	7 (28%)	0	17 (38.64%)	9 (52.94%)	4 (57.14%)	7 (100%)
Prior percutaneous coronary intervention	6 (46.15%)	3 (12%)	0	6 (13.64%)	3 (17.65%)	1 (14.29%)	4 (57.14%)
Prior coronary artery bypass graft	1 (7.69%)	2 (8%)	0	4 (9.09%)	2 (11.76%)	1 (14.29%)	1 (14.29%)
Prior myocardial infarction (MI)	0	1 (4%)	0	3 (6.82%)	0	0	2 (28.57%)
Peripheral arterial disease	5 (38.46%)	1 (4%)	0	4 (9.09%)	8 (47.06%)	0	0
Prior atrial fibrillation	7 (53.85%)	23 (92%)	7 (87.50%)	40 (90.91%)	14 (82.35%)	5 (71.43%)	7 (100%)
Permanent Pacemaker (PPM)	0	0	1 (12.50%)	3 (6.82%)	0	0	2 (28.57%)
Left ventricular ejection fraction, %	55 (50–60)	55 (40–55)	52.5 (42.5–55)	55 (45–60)	55 (45–60)	50 (45–55)	45 (25–60)
NYHA * Class I–II	1 (7.69%)	4 (16%)	2 (25%)	10 (22.7%)	1 (5.88%)	0	0
NYHA * Class III–IV	12 (92.3%)	21 (84%)	6 (75%)	34 (77.2%)	16 (94.1%)	7 (100%)	7 (100%)
CHA2DS2-VASc score **	4 (3–5)	4 (4–5)	4 (3–5)	4 (3–5)	4 (3–7)	4 (3–5)	4 (3–5)
HAS-BLED score *** Score 0	0	1 (4%)	0	1 (2.27%)	0	0	0
Score 1–2	7 (53.8%)	13 (52%)	4 (50%)	26 (59%)	6 (35.3%)	3 (42.8%)	1 (14.3%)
Score ≥ 3	6 (46.1%)	11 (44%)	4 (50%)	17 (38.6)	11 (64.7%)	4 (57.1%)	6 (85.7%)
Never smoked	5 (38.46%)	15 (60%)	3 (37.5%)	11 (25%)	7 (41.18%)	3 (42.86%)	4 (57.14%)
current SMOKER, but unknown frequency	0	0	0	2 (4.55%)	1 (5.88%)	0	0
Former smoker	0	0	0	3 (6.82%)	0	0	1 (14.29%)
Unknown	8 (61.54%)	10 (40%)	5 (62.5%)	28 (63.64%)	9 (52.94%)	4 (57.14%)	2 (28.57%)
Creatinine Clearance (mL/min)	44.1 (34–71.7)	63.4 (52.9–73.1)	67.2 (63.8–75.8)	67.6 (52.25–98.8)	70.6 (50.1–83.6)	80.8 (41–87.2)	58.9 (47.8–67.9)
Hemoglobin (g/dL)	12.5 (11.5–13.1)	12 (10.8–13.8)	13.8 (12.1–14.6)	12.25 (10.45–13.8)	11.7 (11.2–13.2)	12.1 (10.8–12.8)	12.4 (11.3–13)
Platelet count (10^9^/L)	263 (229–300)	211 (176–248)	159 (151.5–278.5)	263.5 (215.5–297.5)	215 (174–274)	274 (130–342)	240 (237–315)

* NYHA = New York Heart Association Functional Class; ** CHA2DS2-VASc: congestive heart failure, hypertension, age ≥ 75 years (2 points), diabetes, history of stroke/transient ischemic attack/systemic arterial thromboembolism (2 points), vascular disease, age 65–74 years, and female sex; *** HAS-BLED: hypertension, abnormal renal/liver function, history of stroke, history of bleeding, labile international normalized ratio (due to missing data), age > 65 years, and drug consumption with antiplatelet agents, nonsteroidal anti-inflammatory drugs, or alcohol abuse. DAPT = dual antiplatelet; SAPT = single antiplatelet; DOACs = direct oral anticoagulants. The data are presented as medians (Q1–Q3), numbers, and percentages.

**Table 2:** Procedural characteristics of patients with atrial fibrillation and transcatheter aortic valve replacement treated with different antithrombotic regimens.

	DAPT N = 13	DOAC Only N = 25	Warfarin Only N = 8	SAPT + DOAC N = 44	SAPT + Warfarin N = 17	Triple (DAPT + DOAC) N = 7	Triple (DAPT + Warfarin) N = 7
Trans-femoral	12 (92.31%)	25 (100%)	8 (100%)	44 (100%)	15 (88.24%)	7 (100%)	7 (100%)
Trans-apical	0	0	0	0	1 (5.88%)	0	0
Trans-subclavian	1 (7.69%)	0	0	0	0	0	0
Direct aortic	0	0	0	0	1 (5.88%)	0	0
PCI	0	0	0	4 (9.09%)	0	3 (42.86%)	2 (28.57%)
Mitral ViV	0	0	1 (12.5%)	0	0	0	0
Peripheral intervention	0	1 (4%)	0	0	0	0	0

Mitral ViV = mitral valve in valve; PCI: percutaneous coronary intervention. DAPT = dual antiplatelet; SAPT = single antiplatelet; DOACs = direct oral anticoagulants.

**Table 3:** Characteristics of early post-procedure outcomes in patients with atrial fibrillation and transcatheter aortic valve replacement treated with different antithrombotic regimens.

	DAPT N = 13	DOAC Only N = 25	Warfarin Only N = 8	SAPT + DOAC N = 44	SAPT + Warfarin N = 17	Triple (DAPT + DOAC) N = 7	Triple (DAPT + Warfarin) N = 7
Early MI	0	0	0	0	0	0	0
Conduction disturbances requiring PPM	5 (38.46%)	3 (12%)	1 (12.5%)	4 (9.09%)	4 (23.53%)	0	1 (14.29%)
Requiring ICD	0	0	0	0	1 (5.88%)	0	0
Early Stroke	0	0	1 (12.5%)	1 (2.3%)	0	0	0
Retroperitoneal bleeding	1 (7.69%)	0	0	0	0	0	0
Gastrointestinal bleeding	0	0	0	1 (2.27%)	0	0	0
Genitourinary bleeding	1 (7.69%)	0	0	1 (2.27%)	0	0	0
Other bleeding	0	0	0	0	2 (11.76%)	0	0

MI = myocardial infarction; ICD = implantable cardioverter defibrillator; PPM = permanent pacemaker; DAPT = dual antiplatelet; SAPT = single antiplatelet; DOACs = direct oral anticoagulants.

**Table 4:** Primary outcomes in patients with atrial fibrillation and transcatheter aortic valve replacement treated with different antithrombotic regimens.

	DAPT N = 13	DOAC only N = 25	Warfarin only N = 8	SAPT + DOAC N = 44	SAPT + Warfarin N = 17	Triple (DAPT + DOAC) N = 7	Triple (DAPT + Warfarin) N = 7
Stroke or TIA	2 (15.38%)	3 (12%)	1 (12.5%)	3 (6.8%)	2 (11.76%)	1 (14.29%)	2 (28.57%)
Ischemic Stroke	2	3	0	2	1	1	2
Undetermined Stroke	0	0	0	0	1	0	0
TIA	0	0	1	1	0	0	0
Major Bleeding	0	1 (4%)	2 (25%)	5 (11.36%)	0	0	0
GI Bleeding	0	1	2	3	0	0	0
Intraocular Bleeding	0	0	0	1	0	0	0
Genitourinary Bleeding	0	0	0	1	0	0	0
Mortality	5 (38.46%)	2 (8%)	2 (25%)	3 (6.82%)	7 (41.18%)	1 (14.29%)	4 (57.14%)

TIA = transient ischemic attack; GI = gastrointestinal. DAPT = dual antiplatelet; SAPT = single antiplatelet; DOACs = direct oral anticoagulants.

## Data Availability

The data presented in this study are available on request from the corresponding author after institutional approval to share the data, the data are not publicly available due to privacy restrictions.

## Abbreviations

AF: atrial fibrillation. TAVR: transcatheter aortic valve replacement. OAC: oral anticoagulant. SAPT: single antiplatelet therapy. DOAC: direct oral anticoagulant. DAPT: dual antiplatelet therapy. TIA: transient ischemic attack. CAD: coronary artery disease. PCI: percutaneous coronary intervention. PAD: peripheral arterial disease. CHA2DS2-VASc: congestive heart failure, hypertension, age ≥ 75 years, diabetes mellitus, stroke, vascular disease, age 65–74 years, and sex category (female). VARC-2: Valve Academic Research Consortium-2. REDCap: Research Electronic Data Capture.
